# An efficient electrochemical sensing of hazardous catechol and hydroquinone at direct green 6 decorated carbon paste electrode

**DOI:** 10.1038/s41598-021-93749-w

**Published:** 2021-07-23

**Authors:** K. Chetankumar, B. E. Kumara Swamy, S. C. Sharma, S. A. Hariprasad

**Affiliations:** 1 Department of P.G. Studies and Research in Industrial Chemistry, Kuvempu University, Jnanasahyadri, Shankaraghatta, 577451 Shivamogga, Karnataka India; 2 National Assessment and Accreditation Council (NAAC), Naagarabhaavi, Bengaluru, 560072 Karnataka India; 3 Jain University, Bengaluru, 560069 Karnataka India; 4 School of Energy Science and Engineering, Indian Institute of Technology Guwahati, Guwahati, India

**Keywords:** Natural hazards, Chemistry

## Abstract

In this proposed work, direct green 6 (DG6) decorated carbon paste electrode (CPE) was fabricated for the efficient simultaneous and individual sensing of catechol (CA) and hydroquinone (HY). Electrochemical deeds of the CA and HY were carried out by cyclic voltammetry (CV) and differential pulse voltammetry (DPV) at poly-DG6-modfied carbon paste electrode (Po-DG6-MCPE). Using scanning electron microscopy (SEM) studied the surface property of unmodified CPE (UCPE) and Po-DG6-MCPE. The decorated sensor displayed admirable electrocatalytic performance with fine stability, reproducibility, selectivity, low limit of detection (LLOD) for HY (0.11 μM) and CC (0.09 μM) and sensor process was originated to be adsorption-controlled phenomena. The Po-DG6-MCPE sensor exhibits well separated two peaks for HY and CA in CV and DPV analysis with potential difference of 0.098 V. Subsequently, the sensor was practically applied for the analysis in tap water and it consistent in-between for CA 93.25–100.16% and for HY 97.25–99.87% respectively.

## Introduction

Dihydroxybenzene (DHB) isomers like catechol (CA) and hydroquinone (HY) are extensively utilized in the agronomic practices and chemical industry, such as tanning, plastic, pesticides, dyes, rubber, pharmaceuticals and so forth^1–6^. Nevertheless, DHB is venomous and hard to degrade, menacing the environment and crippling animal and human health, it leads to incurring the harm to kidney and liver function affected by CA and HY have also been reported^7–12^. In addition, simultaneously sensing of CA and HY is tough because they mutual interfere with each other during their identification because of their similar structures, properties and coexistence^13, 14^. Consequently, the fabrication of simple, selective, speedy, accurate and sensitive analytical method was needed for the detection and quantifying of CA and HY^15, 16^. Up to now, numerous techniques have been testified for sensing of CA and HY, such as spectrophotometry, capillary electrochromatography (CEC), high-performance liquid chromatography (HPLC), gas chromatography/mass spectrometry (GC/MS), fluorescence (FL, chemiluminescence (CL) and electroanalytical method^17–24^. Amongst, the electrochemical analysis has owing prominent in current years to its advantages like simplicity, portability, low cost, time saving, convenient operation, speedy response, as well as high efficiency, sensitivity and selectivity^25–30^.

**Scheme 1:**
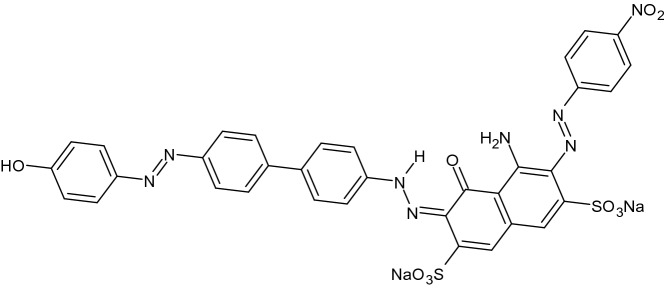
Structure of Direct green 6.

In current decade, polymer modified electrodes (PMEs) have accomplished more attention due to their homogeneity, reproducibility, good constancy and strong adherence to electrode surface^31–34^. Direct green 6 (DG6) mainly utilized in fibers, soap, leather, regenerated cellulose fibers film shading, colouring of paper and also employed in the production of colour pigment sediment^35, 36^ (Scheme 1). The fabrication of the modified electrode was carried out by electropolymerization of DG6 using CV technique and applied for the sensing of CA and HY in presence of biological pH 7.4.

## Experimental section

### Equipment and chemicals

Voltammetric analysis was tested by CV and DPV performances expending a traditional three-electrode cell of CH Instrument-660 (CHI-660c) electrochemical workstation. UCPE and Po-DG6-MCPE were employed as working, saturated calomel electrode (SCE) as a reference and a platinum wire as a counter electrode individually.

HY and CA procured from Sigma-Aldrich and standard solutions (25 × 10^–4^ M) was prepared in double distilled water. DG6, sodium dihydrogen phosphate (NaH_2_PO_4_), disodium hydrogen phosphate (Na_2_HPO_4_) were gained from Merck chemicals of AR grade and all aqueous solution were prepared with double distilled water. All the reagents are utilized in this study with analytical grade and used as received.

## Results and discussion

### Preparation of UCPE and Po-DG6-MCPE

The UCPE was fabricated by blending of graphite powder and silicone oil in the ratio 70:30 (w/w) for about 30 min and get homogeneous mixture. The gotten blend was then filled with homemade Teflon cavity consuming 3 mm internal diameter and cooper wire was utilized for the electrical contact. The pre-treated carbon paste electrode (PCPE) was constructed by electrochemical oxidised by cycling the potential between − 0.6 to 1.0 V in 0.1 M NaOH with speed rate of 0.05 V s^–1^ at 10 multiple cycle.

**Figure 1:**
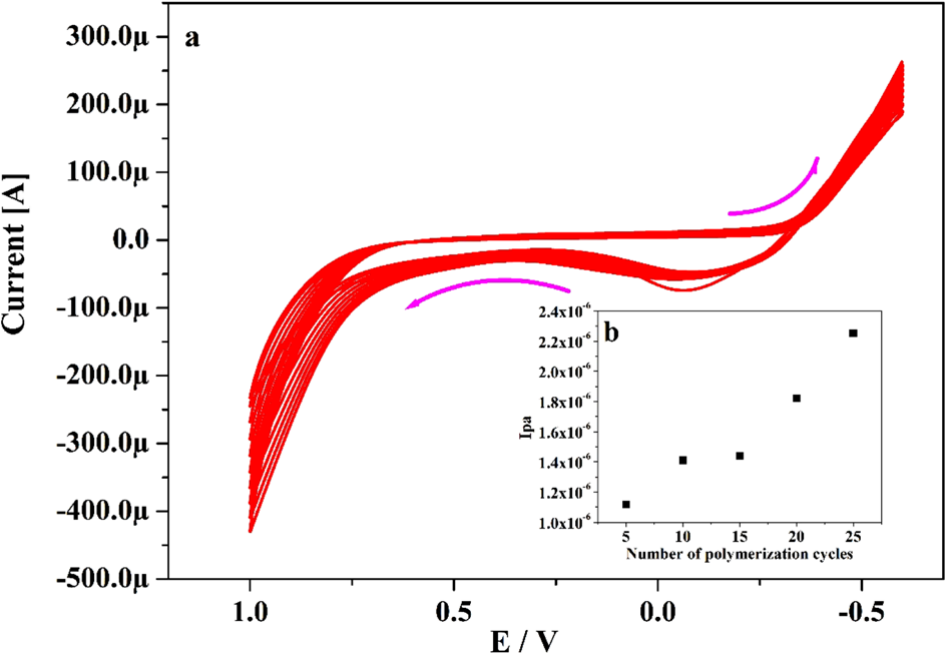
(**a**) Electropolymerization of 1.8 mM DG6 on the surface of UCPE in presence of 0.1 M NaOH with speed rate 0.05 V s^−1^ for 10 multiple cycles. (**b**) Graph of Ipa versus a number of varied electropolymerization cycles.

Electro-polymerisation manner was applied for the constructed fabricated electrode. DG6 (1.8 mM) was carried on the surface of UCPE using cyclic voltammetry in the existence of 0.1 M NaOH (supporting media) and cyclic the potential scanned between − 0.6 to 1.0 V with speed rate 0.05 V s^–1^ for 10 polymerization cycles as elucidates in Fig. 1a. As witnessed from the figure, the peak current gradually boosted with increasing the polymer cycles this endorses the growth of polymeric films on UCPE^37^. The deposition of DG6 on UCPE was carried by changing the polymer series 5 to 25 cycles (Fig. 1b) and applied to identify the electrochemical reactions towards CA in 0.2 M PBS of pH 7.4. By perceiving the Fig. 1b, the Ipa was enhanced upto ten cycles and after 15 cycles it decreases slightly and sudden increment in Ipa for 20 and 25 cycles. Therefore, ten cycles of electro-polymerization were selected and optimised for the fabrication of modified electrode.

### Characteristics and surface property of Po-DG6-MCPE

**Figure 2:**
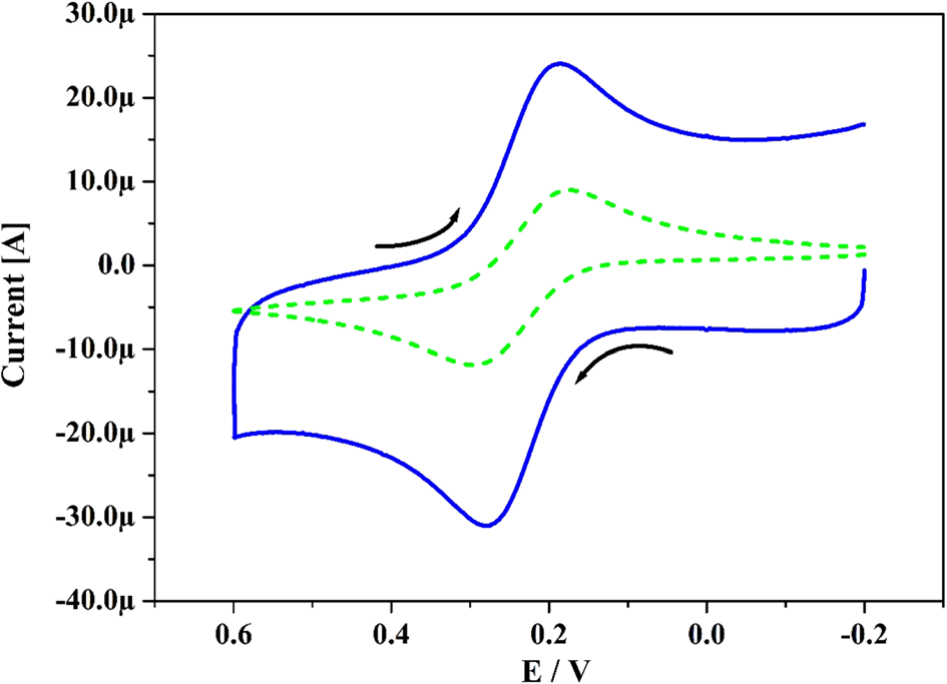
CVs recorded for 1 mM K_4_[Fe(CN)_6_] at UCPE (scattered line) and Po-DG6-MCPE (hard line) at speed rate of 50 mV s^−1^ using 1 M KCl.

The characteristic property of Po-DG6-MCPE was verified using K_4_[Fe(CN)_6_] (25 × 10^–3^ M) as redox probe in 1 M KCl (supporting media). Figure 2 signifies the gotten cyclic voltammograms (CVs) for UCPE (scattered line) and hard-line curve for the Po-DG6-MCPE with speed rate 0.05 V s^–1^. At UCPE, it implies small peak current and in the similar form Po-DG6-MCPE reveals superior boost in peak current than UCPE. Therefore, this development in peak current donated substantial increase in electroactive surface area. This active surface area of the electrode was identified using Randles–Sevick’s Eq. (1) ^38^. Compared to Po-DG6-MCPE (0.061 cm^2^) the UCPE (0.030 cm^2^) was consumed less electroactive surface area. The approximate adhered modifier thickness or surface average concentration on UCPE was calculated by utilising Eq. (2) ^39^ and got at 0.271 × 10^−10^ M cm^−2^.

**Figure 3:**
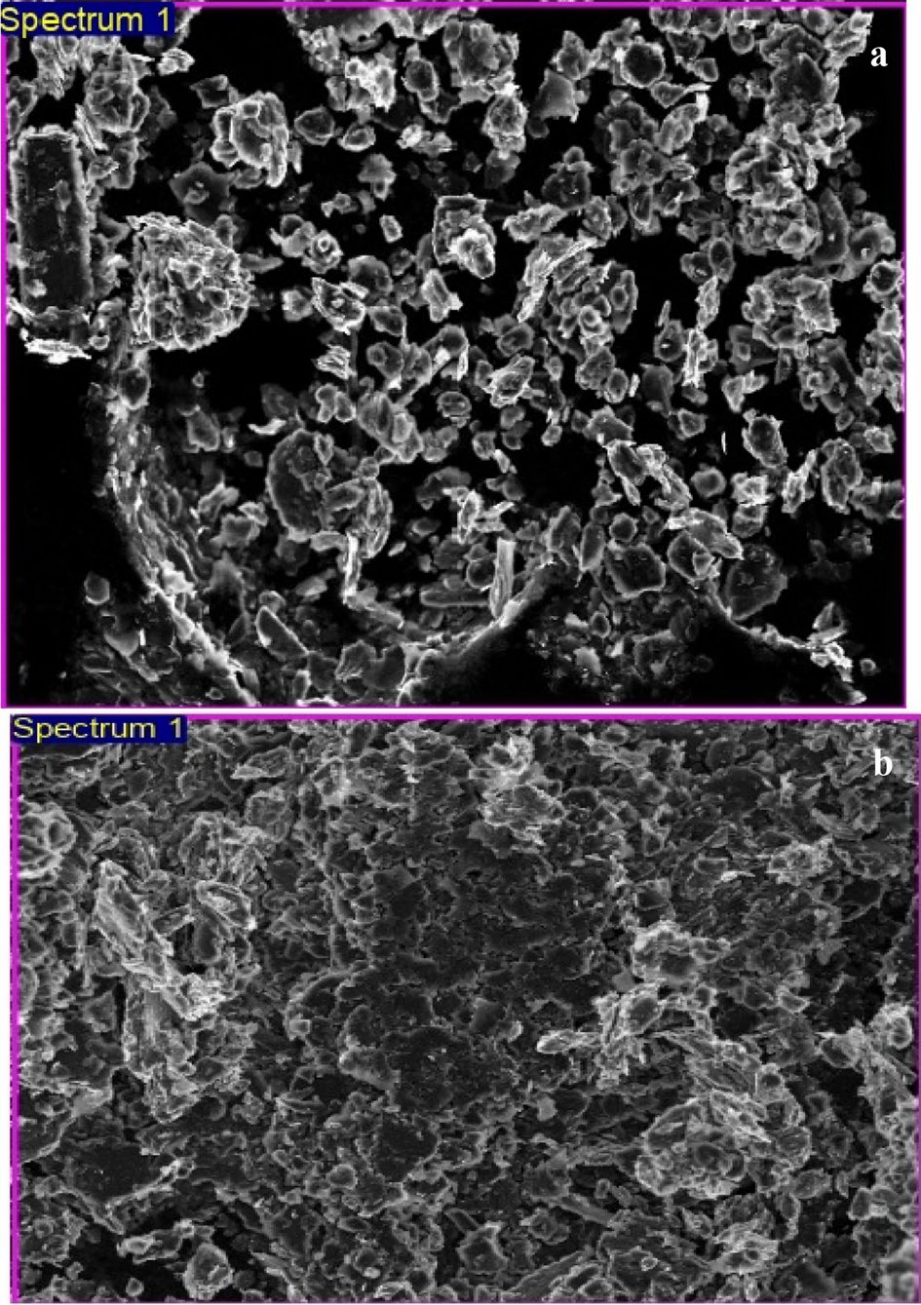
SEM images for (**a**) BCPE and (**b**) Po-DG6-MCPE.

The surface changes of before and after fabrication of UCPE was characterized by SEM using ZEISS Ultra-55. The Fig. 3a and b exposes the achieved SEM pictures of UCPE and Po-DG6-MCPE. In UCPE, the films portray asymmetrical designed flakes of CPE. After modification of DG6, the film was uniformly associated on the superficial of UCPE. This morphological variation on the electrode surface lead to rise in the electro-active surface area and favoured good electrocatalytic accomplishment^40, 41^.

1 $$Ip=2.69\times{10}^{5} {n}^{3/2}{AD}^{1/2}Co \, {\nu }^{1/2}$$

2 $$Ip={n}^{2}{F}^{2}A\Gamma \nu /4RT$$

where *A* is the area of working electrode (cm^2^), *C*_*0*_ is the concentration of the electroactive species (mol/cm^3^), *n* is the electrons transformed, *ν* is the sweep rate (V/s), *D* is the diffusion coefficient (cm^2^ s^−1^), *Ip* is the peak current, *Γ* (M/cm^2^) is the surface average concentration and R, F, T are the physical constants.

### Electrocatalytic performance of CA at Po-DG6-MCPE

**Figure 4:**
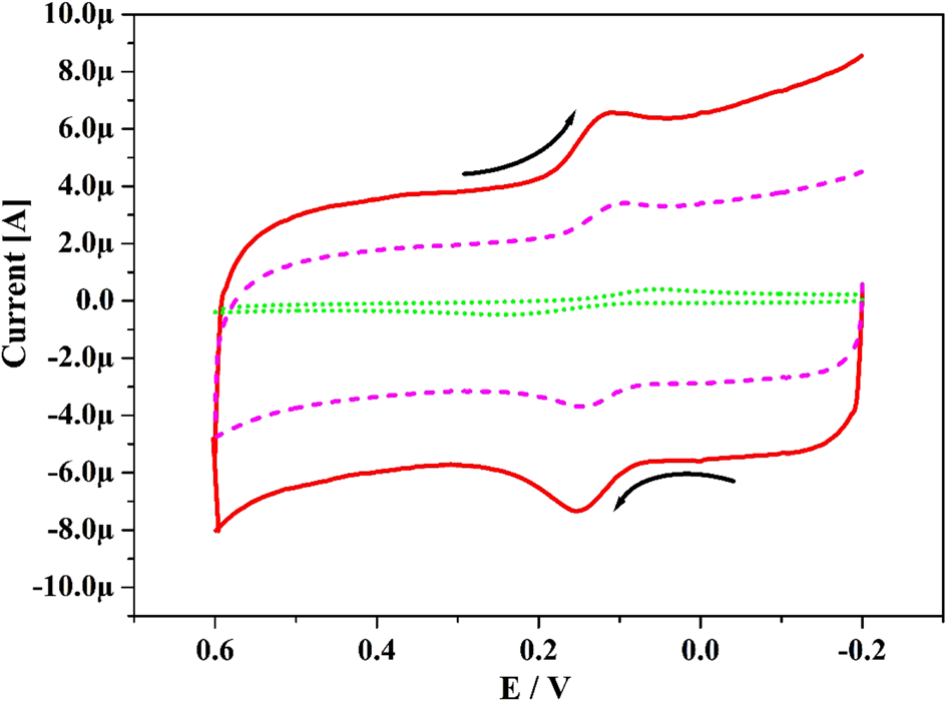
CVs curve for 10 µM CA at UCPE (scattered line) and Po-DG6-MCPE (dashed line) with speed rate 0.05 V s^−1^ using 0.2 M PBS of pH 7.4.

**Scheme 2:**
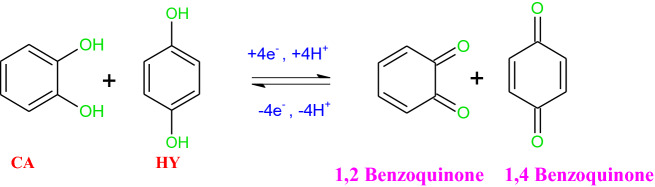
Redox mechanism of CA and HY.

The sensing proficiency of Po-DG6-MCPE towards the CA was scanned by CV technique as most sensitive and precise voltammetric technique. In Fig. 4 the dotted line (green), dashed line (pink) and hard line (red) depicts CVs for UCPE, PCPE and Po-DG6-MCPE for CA (10 μM) in 0.2 M PBS with speed rate of 0.05 V s^−1^. The UCPE provides CVs with reduced and broad response and at PCPE it gave voltammetric response with poor response. Compared UCPE and PCPE the designed electrode signifies ultimate enrichment in peak current with very precise sensitivity with sharp peaks. The peak potential difference (ΔEp = Epa-Epc) was acquired at 0.153 V (BCPE), 0.095 V (PCPE) and 0.017 V (Po-DG6-MCPE) individually. At tailored electrode the electron transfer was easier than UCPE and PCPE, because where ΔEp value is inferior and transfer of electron rate will be greater. Therefore, the Po-DG6-MCPE accomplished as a good sensor and prominent for the analysis of CA. The redox response of CA was portrayed in Scheme 2.

### Effect of speed rate and concentration study of CA at Po-DG6-MCPE

**Figure 5:**
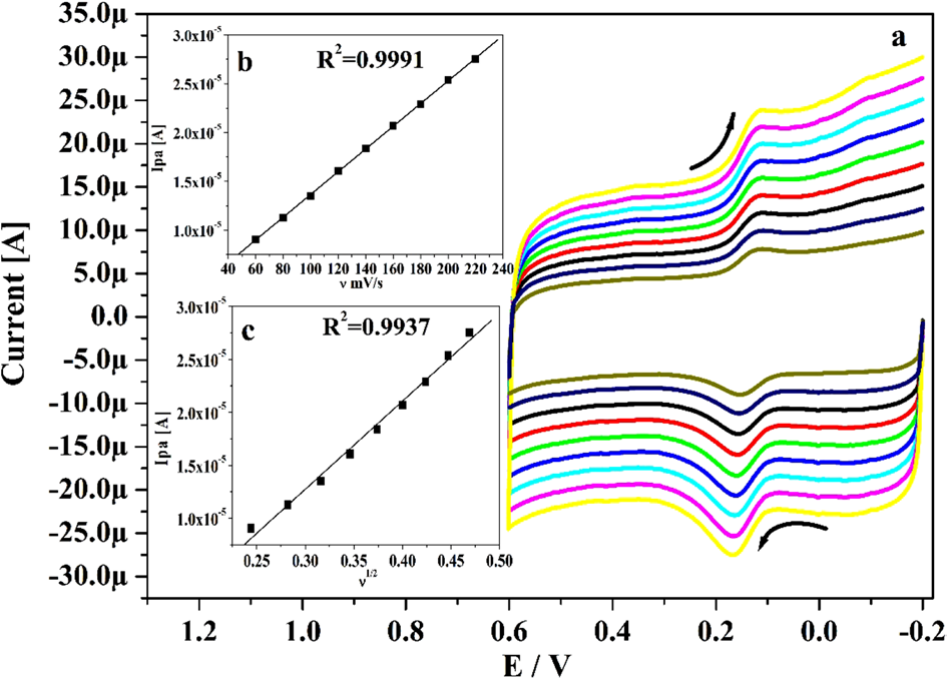
(**a**) CVs for 10 µM CA at Po-DG6-MCPE with changed speed rates (0.06–0.22 V s^−1^) using 0.2 M PBS of pH 7.4. (**b**) Plot of Ipa versus speed rate. **(C)** Plot of Ipa versus square root of speed rate.

The Po-DG6-MCPE process was assessed by changing the speed rate. Figure 5a elucidated the CVs for CA (10 μM) in presence of supporting media with altered speed rates. As seeming in figure, the redox (oxidation and reduction) peak current of CA was successively boosted as increase in the speed rate (60 to 220 mV s^−1^) and minute move of their peak potential to positive and negative side. The linear correlation between anodic peak current (Ipa) versus speed rate (ν) and Ipa versus square root of the speed rate was drawn in Fig. 5b and c. The gotten plot gave very fine straight line with correlation coefficient value (R^2^) was initiate at 0.9991 and 0.9937 correspondingly. Consequently, by spotting the overhead practical result the kinetic property of Po-DG6-MCPE was originated at adsorption controlled process^42, 43^. Heterogeneous rate constant (k^0^ in s^−1^) were computed by Eq. 3 and attained results are tabulated in Table 1.

3 $$\Delta {\text{Ep }} = {\text{ 2}}0{\text{1}}.{\text{39 log }}\left( {\nu /{\text{ k}}^{0} } \right) - {\text{ 3}}0{\text{1}}.{\text{78}}$$

where, k^0^, ΔEp, ν, is the heterogeneous rate constant in s^-1^, difference in peak potential, speed rate respectively.

**Figure 6:**
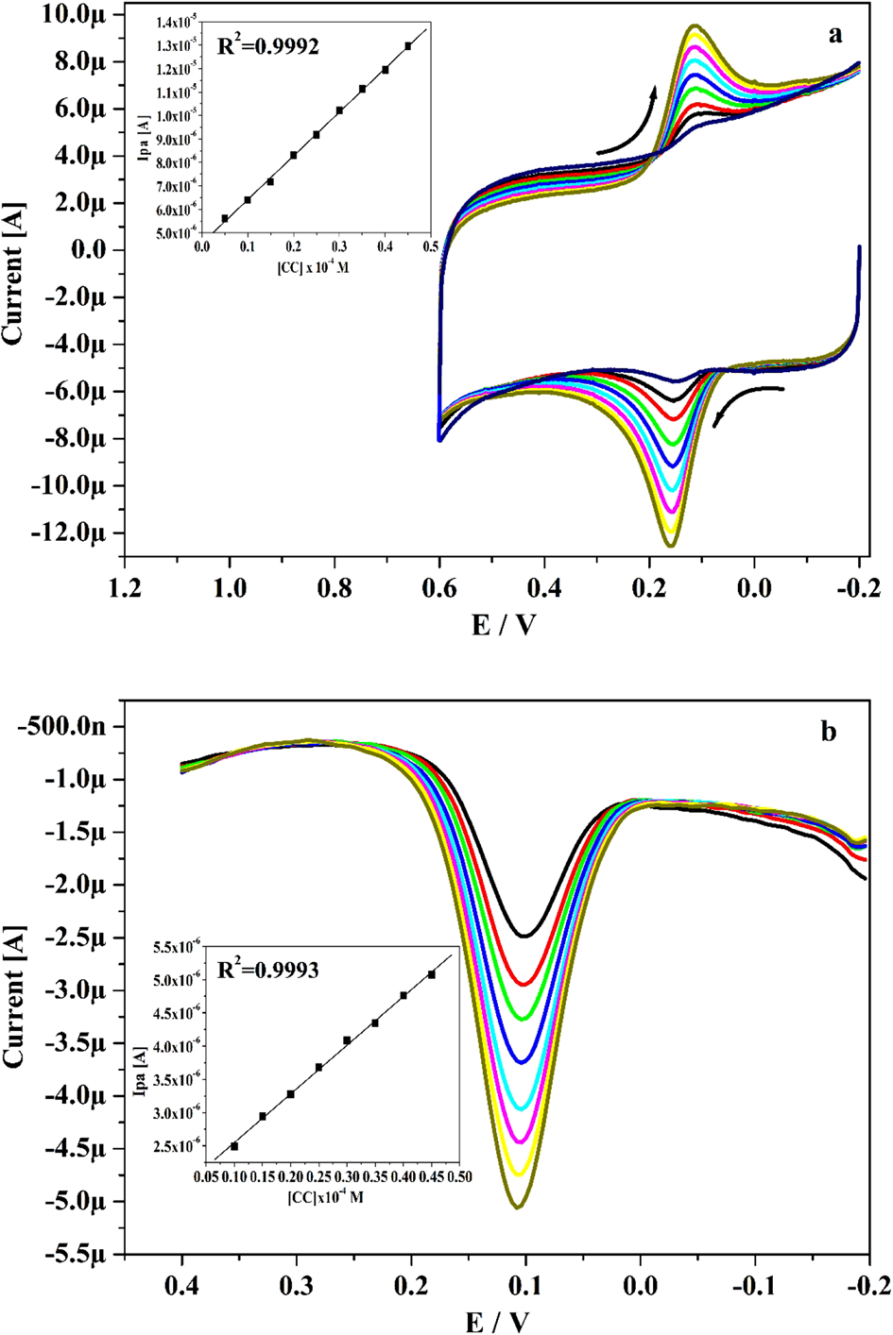
(**a**) Obtained CVs for CA at Po-DG6-MCPE with varied concentration (5–45 µM) in 0.2 M PBS (pH 7.4) with speed rate of 0.05 V s^−1^ and inset figure is graph of Ipa versus concentration of CA. (**b**) DPVs recorded for varied concentration of CA (10–45 µM) at Po-DG6-MCPE and inset figure is plot of Ipa versus concentration of CA.

The revealing of detection sensitivity at modified electrode was assessed by utilizing CV and DPV performances. Figure 6a and b epitomizes the obtained CVs (5–45 μM) and DPVs (10–45 μM) for altered concentrations of CA in presence of pH 7.4 with speed rate of 0.05 V s^−1^. These figures evidently portray that the peak current was boosted noticeably when the analyte concentration was rises. Inset Fig. 6a and b indicates the correlation between Ipa and concentration of analyte with good linearity values of R^2^: 0.9992 and 0.9993. Utilizing the slope value (M) and standard deviation (S) of the Ipa (acquired from inset Fig. 6a and b) assessed the LLOD and LOQ employing Eqs. (4) and (5)^44^ for CA and gotten at 0.09 and 0.29 μM separately.

4 $${\text{LLOD}} = {\text{3S}}/{\text{M}}$$

5 $${\text{LOQ}} = {\text{1}}0{\text{S}}/{\text{M}}$$

**Table 1:** Heterogenous rate constant for CA and HY at Po-DG6-MCPE.

Scan rate (mV s^−1^)	ΔEp (mV)		Heterogeneous rate constant (k^0^) s^−1^	
	CA	HY	CA	HY
60	26	19.7	1.414	1.520
80	38.2	31.7	1.640	1.766
100	46.1	35	1.873	2.126
120	48.9	35.9	2.177	2.526
140	48	36.8	2.566	2.916
160	53	37.8	2.769	3.295
180	57.7	38.5	2.953	3.678
200	60.7	38.9	3.170	4.068
220	65.7	40.7	3.293	4.383
240	–	41.9	–	4.717

### Optimization of pH on CA at Po-DG6-MCPE

**Figure 7:**
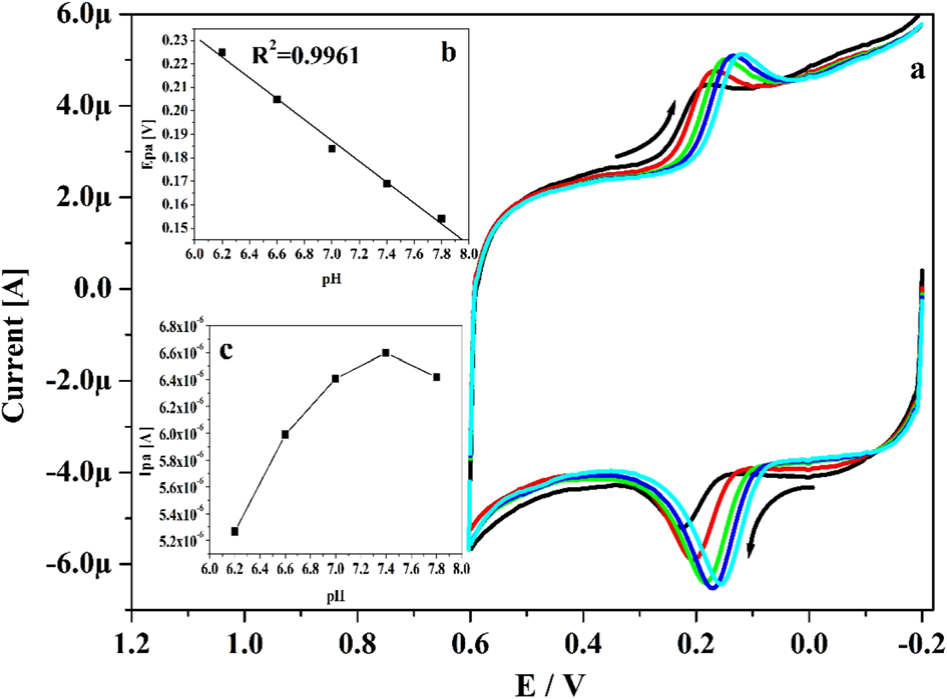
(**a**) Recorded CVs for 10 µM CA at Po-DG6-MCPE in presence of altered pH. (**b**) Plot of Epa versus pH. (**c**) Plot of Ipa vs. pH.

The choice of supporting electrolytes plays major part in the electrochemical study. Figure 7a and exposes the acquired CVs for CA (10 μM) in existence of 0.2 M PBS of changed pH (6.2, 6.6, 7.0, 7.4, 7.8) with speed rate 0.05 V s^−1^ at Po-DG6-MCPE. As witnessed in Fig. 7a, it obviously exposed that as the pH solution was changed then the peak potential of CA was moved to more negative side. This accomplished result was proof for the directly participation of proton in the electrochemical reaction. The linear connection between Epa and varied pH of CA was clarified in inset Fig. 7b. The attained regression eqn. is expressed for CA as Epa (V) = − 0.049 pH + 0.445 (R^2^ = 0.9961). The acquired slope value 49 mV pH^−1^ was very close to the Nernstian theoretical value (59 mV), so it clearly advises the equal number of electron and proton was involved in the electrode reaction^43, 45^. Figure 7c portrays the plot of Ipa versus altered pH solution. By considering maximum peak current and the sensitivity pH 7.4 solution was optimised for further electrochemical exploration.

### Electrocatalytic performance of HY at Po-DG6-MCPE

**Figure 8:**
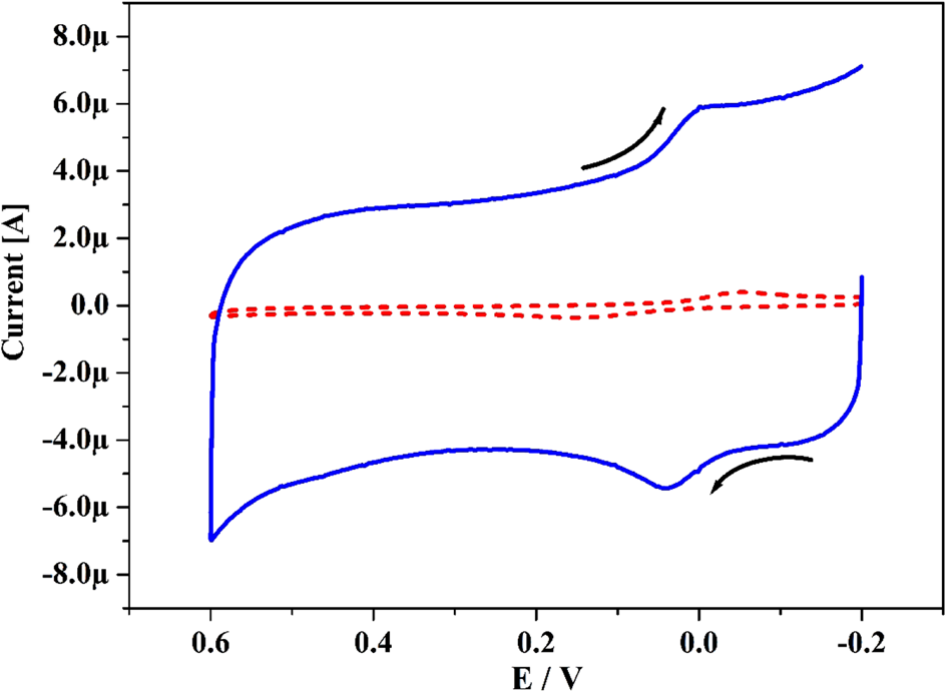
CVs for 10 µM HY in 0.2 M PBS (pH 7.4) at UCPE (scattered line) and Po-DG6-MCPE (hard line) with speed rate of 50 mV s^−1^.

The electrocatalytic sensing of HY was traced by cyclic voltammetry and Fig. 8 elucidates the attained CVs for HY (10 μM) in presence of 0.2 M PBS of pH 7.4 with speed rate 0.05 V s^−1^ at Po-DG6-MCPE. At BCPE (scattered row), it portrays low peak current with poor response because of sluggish electron transfer. Similarly, at established Po-DG6-MCPE (hard row) it provided superior enlargement in redox peak current with very precise sensitivity with sharp than UCPE. The ΔEp value for Po-DG6-MCPE and UCPE was gotten at 0.018 and 0.07 V respectively. Where ΔEp is lower than electron transfer rate will be greater, hence at the constructed electrode electron transfer is easier than UCPE. Thus, the Po-DG6-MCPE achieved as good sensor and prominent for the investigation of HY and redox mechanism was signifying in Scheme 2.

### Impact of speed rate and concentration variation on HY at Po-DG6-MCPE

**Figure 9:**
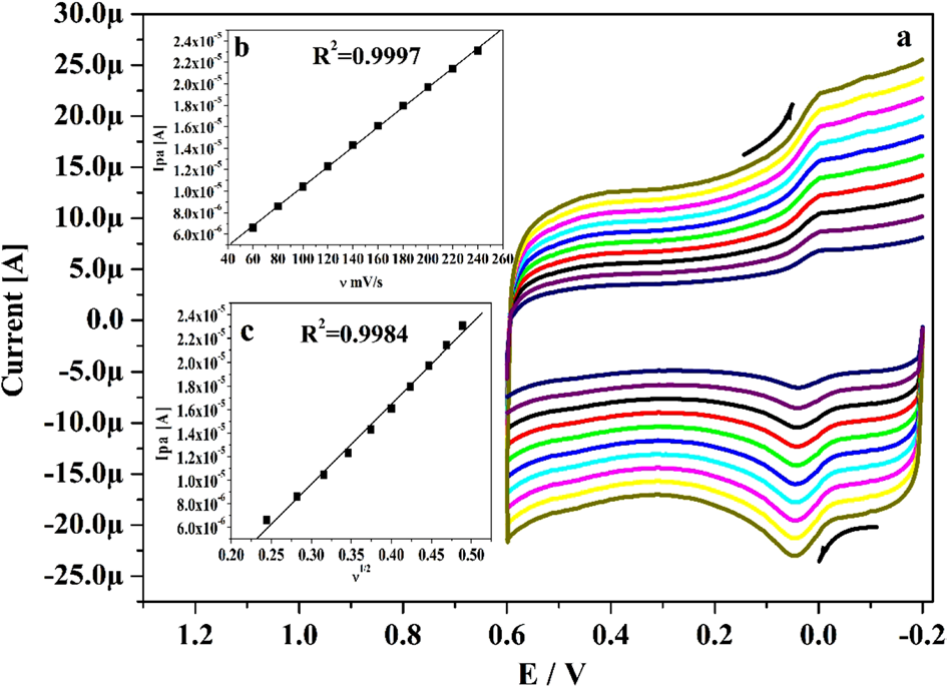
(**a**) CVs for 10 µM HY in 0.2 M PBS (pH 7.4) at Po-DG6-MCPE with varied speed rates (0.06–0.24 V s^−1^). (**b**) Graph of Ipa versus speed rate. **(c)** Graph of Ipa versus square root of speed rate.

The speed rate study offered the essential confirmation about the electrode process. Figure 9a exhibits the gotten CVs for HY in the existence of 0.2 M PBS (pH 7.4) with various speed rate (0.06 to 0.24 V s^−1^). By witnessing the Fig. 9a, as the speed rates elevated the redox peak current is improved subsequently with tiny move in their peak potential to positive and negative potentials. The relationship between Ipa versus speed rate and Ipa versus square root of speed rate was plotted in Fig. 9b and c correspondingly. The plotted graph gave straight linearity with R^2^ value was originate at 0.9997 and 0.9984 respectively and electrode process was governed by adsorption controlled process.

**Figure 10:**
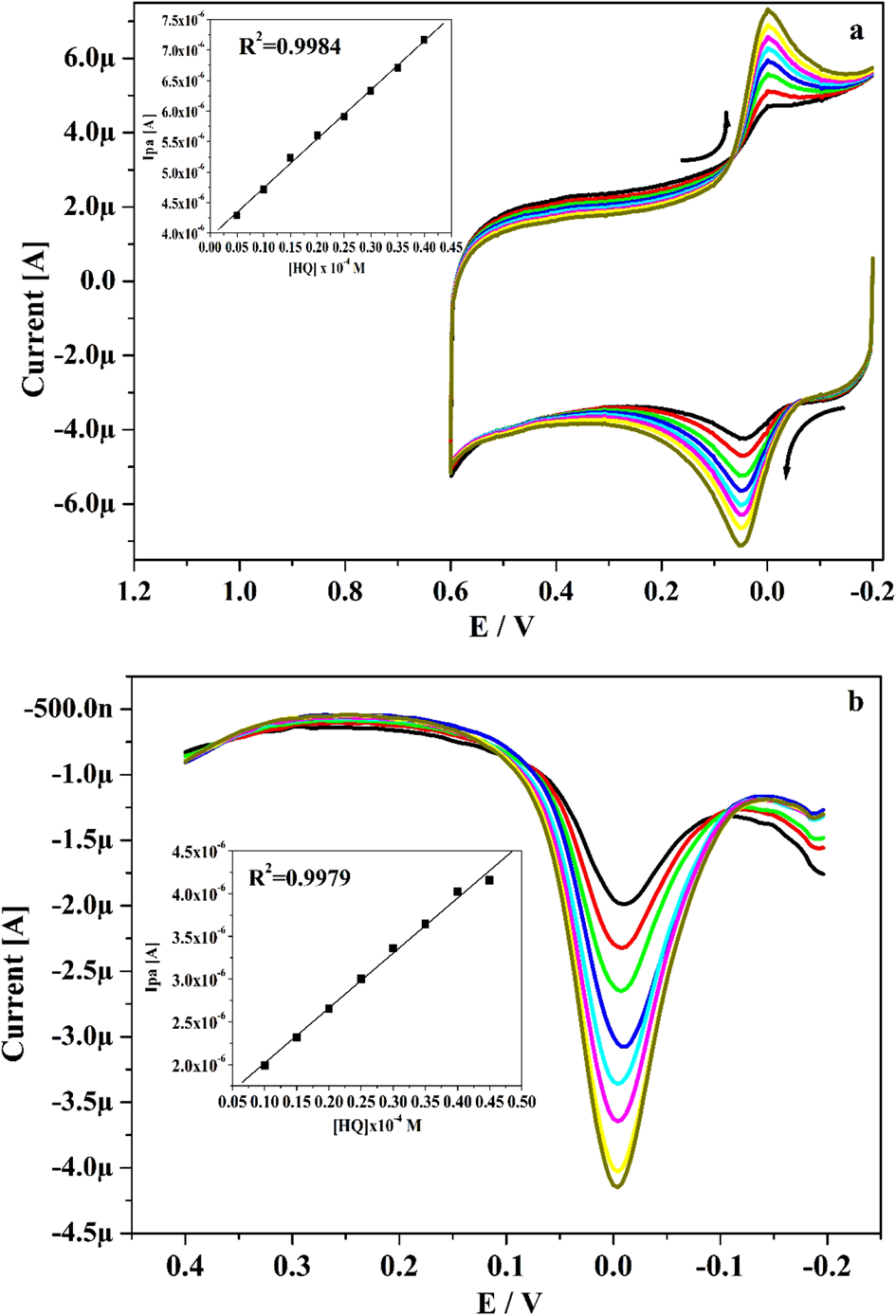
(**a**) CVs curve for altered concentration of HY (5–40 µM) at Po-DG6-MCPE in 0.2 M PBS (pH 7.4) with speed rate of 0.05 V s^−1^ and inset figure is plot of Ipa versus concentration of HY. (**b**) DPVs curve for varied concentration of HY (10–45 µM) at Po-DG6-MCPE and inset figure is plot of Ipa versus concentration of HY.

The LLOD and LOQ was calculated utilizing Eqs. (4) and (5) for HY by applying the CV and DPV performance. Figure 10a and b epitomizes the obtained CVs (5–40 μM) and DPVs (10–45 μM) for altered concentration (5–40 μM) of HY in existence of pH 7.4 with speed rate of 0.05 V s^−1^. As noticed in Fig. 10a and b, the oxidation peak current of HY was growths linearly as the concentration raises and peak potential tiny swing towards negative and positive direction. The inset Fig. 10a and b portrays the connection between concentration of HY and Ipa and it gave very adequate linearity with R^2^ value 0.9984 and 0.9979. The LLOD and LOQ was establish at 0.11 and 0.36 μM correspondingly. The Po-DG6-MCPE offered low LLOD for CA and HY than other fabricated sensor and displayed in Table 2.

**Table 2:** Comparison of LLOD at Po-DG6-MCPE with other reported modified electrodes for the determination CA and HY.

Working electrode	Limit of detection (µM)		Reference
	CC	HQ	
COF/MCPE	0.46	0.31	^2^
NCNTFs/GCE	0.12	0.17	^7^
PIL-MCNs/CS/GCE	0.62	1.39	^8^
CoFe_2_Se_4_/PCF	0.15	0.13	^9^
Activated GSEC	0.1	0.1	^15^
Pt/C_60_/PGE	2.97	2.19	^16^
Fluorescent polymer nanoparticles (FPNs)	0.33	0.21	^51^
CA-GCE	0.23	0.46	^52^
3D sulfur/nitrogen co-doped graphene	0.28	0.15	^53^
DCIL	0.40	0.31	^54^
Co_3_O_4_/MWCNTs/GCE	8.5	5.6	^55^
PPGE	1.32	1.17	^56^
PCFCuNP/GE	0.53	1.1	^57^
GCE/MtH-NH_2_	0.65	–	^58^
Poly(vanillin)/MCPE	0.95	0.99	^59^
GDYO/GCE	0.2	0.3	^60^
Po-DG6-MCPE	0.09	0.11	Present work

### Simultaneous sensing capability of Po-DG6-MCPE

**Figure 11:**
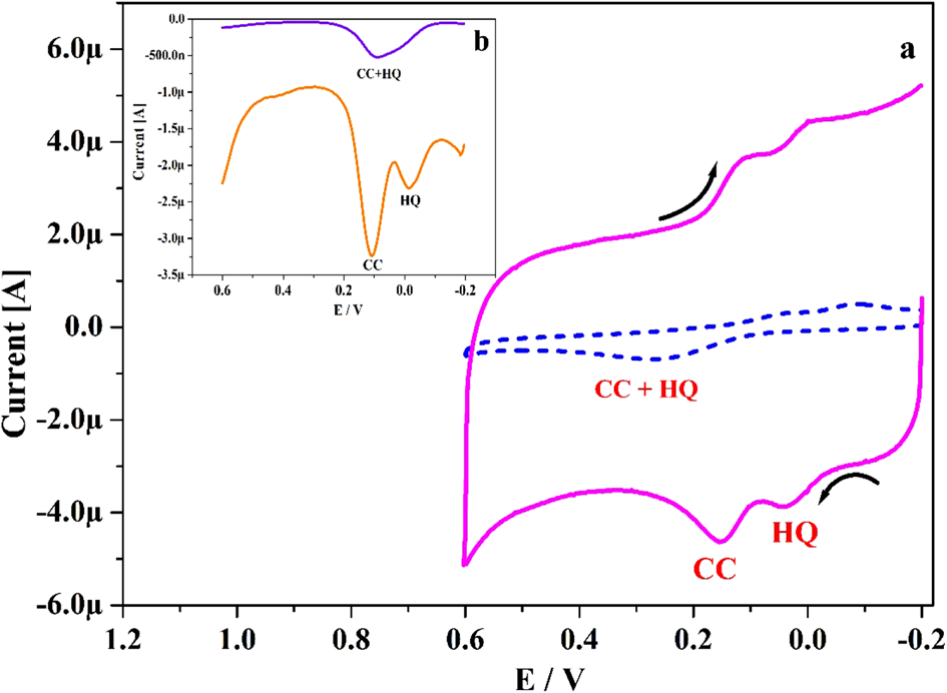
(**a**) CVs curve for simulataneous sensing of CA and HY (10 µM) in 0.2 M PBS of pH 7.4 at UCPE (scattered line) and Po-DG6-MCPE (hard line) with speed rate 0.05 V s^−1^. (**b**) DPVs for simulataneous sensing of CA and HY (10 µM) in 0.2 M PBS of pH 7.4 at UCPE (blue hard line) and Po-DG6-MCPE (saffron hard line).

The simultaneous revealing of CA and HY was very challenging in mixed solution at UCPE. Because in the mixed solution they are overlapped each other due to their approximately same oxidation potential^46^. Thus, to validate the potentiality of designed electrode for the simultaneous exploration of CA and HY. Figure 11a discloses the gotten CVs curve for CA and HY (10 μM) in existence of 0.2 M PBS (pH 7.4) with speed rate 0.05 V s^−1^. The UCPE (scattered line) was failed to depicts the two oxidation peaks but gave only one oxidation peaks located at 0.258 V. Moreover, the fabricated Po-DG6-MCPE (solid streak) clearly verified the two separated oxidation peaks for CA and HY (0.15 and 0.03 V) individually with magnificent development in peak current compared to UCPE. Figure 11b exposes the DPVs for CA and HY in existence of 0.2 M PBS (pH 7.4). Here also (Fig. 11b), at UCPE it fails to separate the oxidation peaks for CA and HY but at the designed electrode it clearly depicts two well distinguish sharp peaks (0.105 and 0.014 V) with improvement in their peak current than UCPE. Therefore, the designed Po-DG6-MCPE was excellent capability for the simultaneous recognition of CA and HY.

### Selectivity and stability of CA and HY at Po-DG6-MCPE

**Figure 12:**
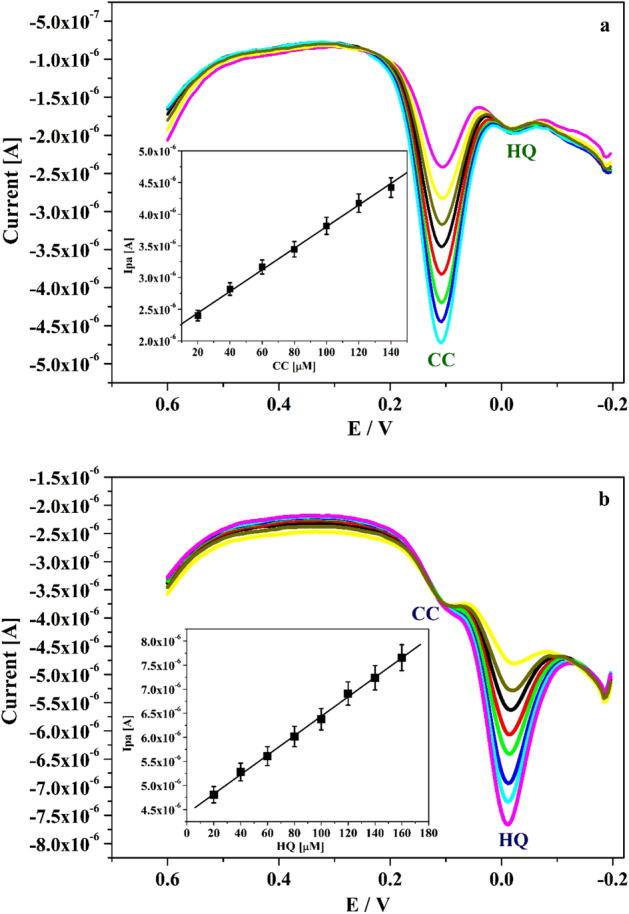
(**a**) DPVs of CA with changed concentration (20–160 µM) in presence of HY (20 µM) and inset figure is graph of Ipa versus concentration of CA. (**b**) DPVs curve for varied concentartion of HY (20–160 µM) in presence of CA (20 µM) in 0.2 M PBS (pH 7.4) at Po-DG6-MCPE.

The efficiency and selectivity detection of CA and HY was testified at Po-DG6-MCPE by applying the DPV technique. Figure 12a represents the tracing of CA by kept the concentration of HY (20 μM) was constant. As we perceived, the peak current of CA was boosted by increasing the concentration of CA in the range 20–160 μM. Inset the Fig. 12a implies the linearity graph of Ipa versus altered concentration of CA. Likewise for HY, the concentration was altered in the range 20 to 160 μM and CA (20 μM) concentration was kept constant and signified in Fig. 12b. By witnessing the above consequence, as the concentration of analyte increased the peak current was boosted gradually but there was no variation in peak potential and peak current of constant analytes. Inset the Fig. 12b displays the linearity relationship of Ipa versus changed concentration of HY. This outcome specifies that the constant analytes are did not interfere in the analysis. Therefore, Po-DG6-MCPE have commendable selectivity and they are tested individually in mixed solution.

**Figure 13:**
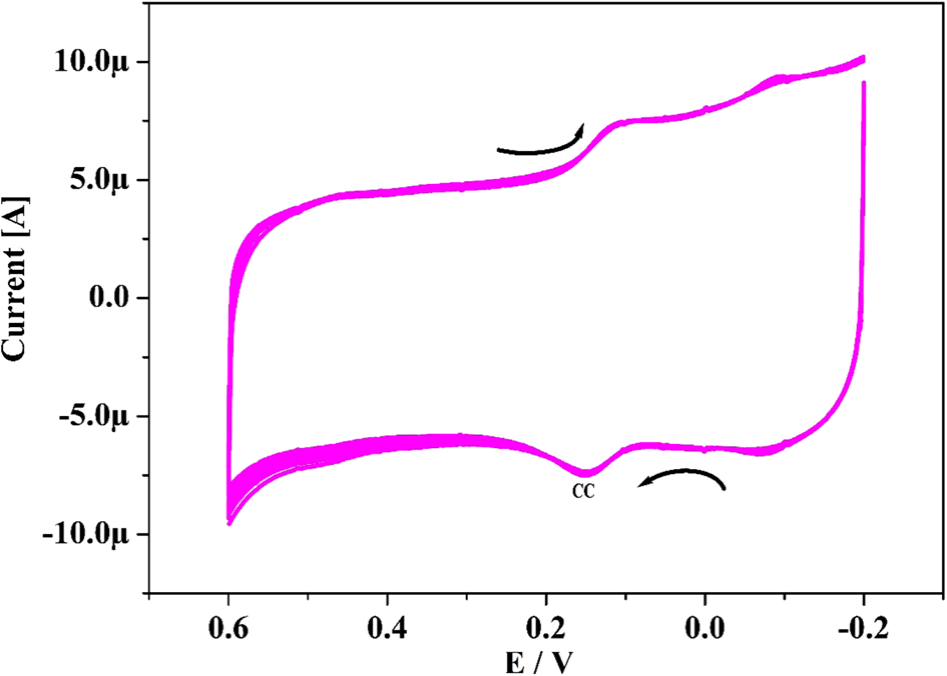
CVs of CA(10 µM) in presence of 0.2 M PBS of pH 7.4 with speed rate 0.05 V s^−1^ at Po-DG6-MCPE for 10 cycles.

The stability of the Po-DG6-MCPE was assessed for CA (10 µM) in presence of 0.2 M PBS for 10 cycles with speed rate of 50 mV s^−1^ by utilizing CV system. As depicted in Fig. 13 the redox current remains steady and after accomplishment 10 multiple cycles the small reductions in their redox current was 4.57%. The percentage degradation was assessed by eqn. % degradation = Ip_n_/Ip_1_^47, 48^, where Ip_1_ and Ip_n_ are the 1st and nth cycle Ipa respectively. The retained steadiness of the Po-DG6-MCPE was 95.43% and this confirms the established electrode have magnificent stability.

### Interference and practical analysis

The anti-interference capability of Po-DG6-MCPE were tested for CA and HY in presence of some coexisting compounds such as resorcinol, uric acid, serotonin, NaCl and KCl and CaCl_2_. There was no considerable interference was perceived for the analysis. Thus, the proposed Po-DG6-MCPE sensor reveals the excellent anti-interference ability.

Finally, the fashioned Po-DG6-MCPE was testified for the practical applicability of CA and HY in a local tap water sample using standard addition method^49, 50^. This advises the recommended electrode exposes satisfactory recovery for each sample addition and attained results are recorded in Table 3. Therefore, this result clarifies the accuracy of constructed electrode for the revealing CA and HY in tap water.

**Table 3:** Recoveries of CA and HY in a local tap water sample at Po-DG6-MCPE.

Samples	Add (μM)		Found (μM)		Recovery (%)	
	CA	HY	CA	HY	CA	HY
Tap water	4	4	3.93	3.89	98.25	97.25
	8	8	7.46	7.99	93.25	99.87
	12	12	12.02	11.73	100.16	97.55
	16	16	15.91	15.96	99.43	99.75

## Conclusion

The proposed work reports Poly-DG6-MCPE was accomplished as a sensor for the detection of CA and HY. The surface morphology of UCPE and Po-DG6-MCPE was characterized by SEM examination. This proposed electrode exposed the strong electrocatalytic activity, high sensitivity, stability and gave improved electron transfer response than UCPE with respect to the oxidation of CA and HY. The impact of pH study, concentration variation and speed rate study was tested at fabricated electrode. The Po-DG6-MCPE involves adsorption-controlled procedure and displayed low detection limit value compared to other reported electrodes. Simultaneous firmness of CA and HY was tracked by CV method. The constructed modified electrode results in good stability, selectivity and offers satisfactory recovery of analytes. Therefore, the mentioned Po-DG6-MCPE was substantial potentiality for the specific and simultaneous investigation and this constructed electrode was utilized for the further analysis of other electro-active biomolecules.
